# Ethical Climate(s), Organizational Identification, and Employees’ Behavior

**DOI:** 10.3389/fpsyg.2019.01356

**Published:** 2019-06-19

**Authors:** Manuel Teresi, Davide Dante Pietroni, Massimiliano Barattucci, Valeria Amata Giannella, Stefano Pagliaro

**Affiliations:** ^1^Laboratory of Social Psychology, Department of Neuroscience, Imaging and Clinical Sciences, Università degli Studi di Chieti-Pescara, Chieti, Italy; ^2^eCampus University, Rome, Italy

**Keywords:** ethical climate, friendship, self-interest, organizational identification, employees’ attitudes

## Abstract

Ethical climate defines what is correct behavior and how ethical issues should be handled within organizations. For this reason, it plays a key role in organizational life. We relied on the social identity approach to compare the effects of two specific ethical climates – an ethical climate of self-interest vs. friendship – on employees’ reactions. In two scenario-based experiments (*N*_1_ = 152 and *N*_2_ = 113), participants were asked to imagine themselves working in an organization described either as characterized by a friendship or a self-interest ethical climate. They completed measures of identification, commitment, perceived organizational morality, turnover intention, recommendation, and the minimum wage they would accept to work for that organization. An ethical climate of friendship predicted better employees’ attitudes and behavioral intentions, and these were mediated by identification with, and commitment to, the organization. In Study 2, participants were less willing to move from an organization characterized by an ethical climate of friendship to a company characterized by an ethical climate of self-interest than vice versa, and asked for more money to accept this new job offer. Results, which confirmed that organizational identification and commitment represent key factors in organizational life, are discussed in terms of practical interventions that promote pro-organizational behavior.

## Introduction

Ethical work climate represents a set of shared formal and informal perceptions of procedures and policies, which shape expectations for ethical behavior (Victor and Cullen, 1987, 1988). In recent years, researchers as well as practitioners have focused their attention on this construct, considering its direct influence both on individual and organizational outcomes and behaviors (for a recent review, see Newman et al., 2017). In particular, when comparing ethical climates that promote prosocial behavior with those suggesting more individualistic behavior, it emerges that the former are more strongly associated with work performance and employees’ positive attitudes and behaviors (e.g., Peterson, 2002; Briggs et al., 2012; Ehrhart and Raver, 2014; Mayer, 2014; Pagliaro et al., 2018). Thus, it seems crucial for organizations to understand the positive and negative consequences of different kinds of ethical climates in order (a) to avoid the associated financial and sociopsychological costs and (b) to rely on those climates that, on the contrary, may increase employees’ positive relationship with the organization and positive behaviors.

In the present research, we attempted to understand how different types of ethical climates predict employees’ (positive and negative) attitudes and behaviors. In doing so, we relied on the social identity approach (Tajfel and Turner, 1979; Turner et al., 1987) to suggest that the effects of (different) ethical climates on employees’ attitudes and behavioral tendencies are driven by identification with the organization (Pagliaro et al., 2018). In particular, we attempted to show that different kinds of ethical climates are related to different levels of organizational identification, which in turn influences the employees’ positive and negative behavioral reactions. Importantly, although previous research has provided preliminary cross-sectional evidence about the associations between different ethical climates and employees’ reactions (Pagliaro et al., 2018), in the present research, we attempted to disentangle the causal link of this relationship. To this aim, we adopted an experimental approach, which to the best of our knowledge has never been considered before in relation to this topic.

## Ethical Climate(s) and Its Importance in Organizations

According to the classical definition by Victor and Cullen (1987), ethical climate may be defined as a set of shared perceptions of procedures and policies, both codified and informal, which shape expectations for ethical behavior within an organization or a company. Olson (1998) elaborated on this definition suggesting that ethical climate provides “the context in which ethical behavior and decision-making occurs” (p. 346). As such, ethical climate tracks behavioral guidelines that help individuals to understand what is acceptable rather than sanctionable within organizations.

Representing a strong group regulation tool (Ellemers et al., 2013), ethical climate becomes central to organizational life as a way to show the core values of the company both internally and externally, to promote identification and commitment with the organization, and to manage deviance (see also Ceschi et al., 2016). Based on its centrality, researchers have investigated the impact of ethical climate on individual’s emotions, perceptions, and behaviors. Indeed, ethical climate has been demonstrated to significantly influence the ethical behavior of employees (e.g., Treviño et al., 1998; Fritzsche, 2000), job attitudes (e.g., job satisfaction; Deshpande, 1996; Schwepker, 2001; Ambrose et al., 2008), commitment to the organization (e.g., Babin et al., 2000), proactive customer service performance (e.g., Lau et al., 2017), turnover intentions (e.g., Mulki et al., 2006), organizational citizenship behaviors (e.g., Leung, 2008; Shin, 2012; Pagliaro et al., 2018; see also Bellini et al., 2019), organizational deviance (e.g., Hsieh and Wang, 2016), corruption (e.g., Gorsira et al., 2018), and a range of counterproductive behaviors including tardiness or absenteeism (Wimbush and Shepard, 1994; Peterson, 2002). To sum up, ethical climate is related to the promotion of positive work behaviors and, on the contrary, to the prevention of deviant work behaviors (for a review, see Newman et al., 2017).

A further route of investigation about ethical climate has focused on its conceptualization and measurement. Although some researchers tend to consider it as a single, holistic construct (e.g., Schwepker, 2001; Shin, 2012; Mayer, 2014), the concept of ethical climate is more often considered as multidimensional (Victor and Cullen, 1988; Babin et al., 2000; DeConinck, 2011). Starting from the original taxonomy by Victor and Cullen (1987, 1988), researchers conceptualized different types of ethical climates, as well as different ways of differentiating between these (e.g., Babin et al., 2000; Schminke et al., 2005; Arnaud, 2010; Schwepker, 2013). Among these theorizations, in the present paper, we are particularly interested in a relevant distinction that can be made based on the individualistic and independent vs. collectivistic and interdependent focus of the ethical climate under consideration. This reflects the distinction that has been made between an ethical organizational climate of *self-interest*, which underlines a more individualistic and independent way of dealing with ethical issues within the organization; and an ethical organizational climate of *friendship*, which, on the contrary, subsumes a collective and interdependent way to deal with the same ethical issues (Cullen et al., 1993).

Even though, in principle, both these climates of friendship and self-interest may have positive effects, we are inclined to believe that the former would be more likely to promote pro-organizational attitudes and behavior, like commitment and organizational citizenship behaviors (OCB), and by contrast to discourage negative tendencies such as turnover intentions. The supposed primacy of the friendship (vs. self-interest) climate on pro-organizational attitudes and behavior relied on the hypothesis that such an interdependent and collective way of dealing with ethical issues within organizations is more likely to promote organizational identification, which should represent the motivational key driving employees’ attitudes and behavior. Indeed, research so far has proposed that organizational identification is related to ethical climate(s) and its consequences, but experimental evidence about this causal linkage is still lacking, at least to the best of our knowledge.

## Organizational Identification and Ethical Climate

The social identity approach is a widespread theoretical framework in the social psychology field, which comprises the social identity theory (Tajfel and Turner, 1979) and its cognitive elaboration, the self-categorization theory (Turner et al., 1987). The core statement of this approach relies on the consideration that whereas in many situations people perceive themselves as unique and independent individuals, in many other contexts, they are inclined to think of themselves in terms of group membership (e.g., as a member of a specific organization). This self-definition in terms of group membership becomes part of the individual’s collective self-concept: As a consequence, group membership, and the connection with one’s group (i.e., social identification), provides individuals with normative guidelines that help them define who they are, how to behave, and which aspects of their group belongingness are particularly important (Ellemers et al., 2004, 2013).

Over the last three decades, there has been an increasing interest in applying the social identity theory to the classical topics of organizational psychology: This has resulted in a new understanding of many organizational dynamics (leader–follower relations, teamwork, job strain, among others; Haslam, 2004). In fact, from Ashforth and Mael (1989, 1996), an extensive bulk of research has documented the link between organizational identification and several aspects of organizational life: This has been recently summarized in a meta-analysis by Lee et al. (2015), which reported significant average correlations between organizational identification and both positive work attitudes (*r* = 0.41; e.g., job involvement, satisfaction, commitment) and behaviors (*r* = 0.29, e.g., in-role and extra-role behaviors, organizational citizenship behaviors) (see also Riketta, 2005; but for a different perspective, see also Conroy et al., 2017). Thus, organizational identification plays a fundamental role in shaping organizational behaviors.

However, despite its centrality in organizational behavior, less attention has been devoted to the connection between organizational identification and ethical climate (DeConinck, 2011; Briggs et al., 2012; DeConinck et al., 2013; Pagliaro et al., 2018). The scarce attention devoted to the empirical investigation of the relationship between ethical climate and organizational identification is surprising, considering also that in the last years researchers have identified the central role of moral/ethical issues for group belonginess and identification (Pagliaro et al., 2011; van Prooijen and Ellemers, 2015). People strive to belong to groups and organizations that are considered moral and honest, and this centrality of organizational morality leads them to commit themselves to the organization (for a review, Ellemers et al., 2013). Thus, a strong ethical climate subsuming a collective and interdependent (vs. an individual and independent) way to behave within the organization should facilitate employee organizational identification and, in turn, should promote pro-organizational attitudes and behavior.

In a recent paper, Pagliaro et al. (2018) highlighted in a sample of employees that the perception of an ethical organizational climate of self-interest was negatively related with organizational identification (and positively related with moral disengagement). This, in turn, seemed to facilitate counterproductive work behaviors and to inhibit organizational citizenship behaviors among employees. Interestingly, and in line with the rationale presented above, an opposite pattern emerged in relation to the perception of an ethical organizational climate of friendship, which was positively related to organizational identification. Then, such an increased level of identification seemed to foster organizational citizenship behaviors and to reduce counterproductive work behaviors among employees. However, although Pagliaro et al. (2018) provided preliminary support to the idea that the effects of different ethical climates on individual’s reactions are driven by the psychological link with the organization (see also DeConinck, 2011), their research remains purely cross-sectional. Thus, no claims about causality should be made. In the present paper, we aim to overcome this issue.

## Overview of the Present Research

The present research has two main objectives. First, it aims to confirm that two kinds of ethical climates differentially influence employees’ identification with the organization and, in turn, employees’ (positive and negative) attitudes and behavioral intentions toward the organization itself. Building on Pagliaro et al. (2018), we focused on two specific ethical climates: an ethical organizational climate of self-interest and an ethical organizational climate of friendship (Cullen et al., 1993). The choice of these two facets of ethical climate was theory driven; in particular, it was based on the core claim of the social identity approach relative to the importance of the psychological link with other ingroup members. Although the authors showed that employees’ perception of an ethical climate of friendship (vs. self-interest) was positively related to identification with the organization, and this increased positive organizational outcomes (i.e., OCB) and decreased negative organizational outcomes [i.e., counterproductive work behaviors (CWB)], their study was purely cross-sectional. Starting from this limitation, the second aim of the present research was to provide evidence about the causal link between the two considered ethical climates on the one hand and the identification with the organization and the subsequent outcomes on the other. To this end, we adopted an experimental approach and also increased the outcomes under investigation to tap a wider range of possible employees’ reactions. In particular, in both the studies reported below, we presented participants with two scenarios describing fictitious organizations, which differ in terms of ethical climates (friendship vs. self-interest) and then assessed their reactions to these descriptions (see below for details).

Based on the above rationale, we hypothesized that in both studies, participants exposed to an ethical climate of friendship compared to those exposed to an ethical climate of self-interest

(Hp1) will identify more strongly with the organization, and

(Hp2) will exhibit more positive attitudes and behavioral intentions toward the organization (in the form of higher commitment, higher perception of organizational morality, lower turnover intention, higher levels of recommendation, and lower acceptable minimum wage).

(Hp3) We further predicted that the effects of the different climates on employees’ attitudes and behavioral intentions would be mediated by organizational identification.

In Study 2, we further allowed participants to take into account a job offer from a new company characterized by the opposite ethical climate of their own organization. In line with our reasoning, we hypothesized that participants exposed to an ethical climate of friendship compared to those exposed to an ethical climate of self-interest

(Hp4) will be less willing to transfer to the new organization characterized by an ethical climate of self-interest (vs. friendship), and

(Hp5) will ask for more money in order to transfer to the new organization characterized by an ethical climate of self-interest (vs. friendship).

The local institution of the first author does not have the formal ethical approval to conduct non-clinical and non-invasive behavioral studies. We, thus, conducted both studies strictly in line with the ethical standards of the 1964 Declaration of Helsinki and with the ethical standards of the national (i.e., Associazione Italiana di Psicologia) and international (APA) referential scientific associations (see below for details).

## Study 1

### Methods

#### Participants and Design

One hundred fifty-two undergraduates were recruited at the beginning of a psychology course (127 females, 24 males, 1 unknown; mean age = 21.07; SD = 2.05) and participated in the study for course credits. Participants were randomly assigned to one of the two conditions resulting from a between-participants design with one factor (ethical climate: friendship vs. self-interest).

#### Procedure

Participants were presented with a paper-and-pencil questionnaire, allegedly investigating the opinion about some aspects of organizational life. In line with the ethical standards of the 1964 Declaration of Helsinki, before taking part in the experiment, participants were informed about any relevant aspect of the study (e.g., methods, institutional affiliations of the researcher); they were informed of the right to refuse to participate in the study or to withdraw consent to participate at any time during the study without recrimination. They then confirmed that they understood the instructions well, accepted the conditions to participate in the study, and filled out the questionnaire. All participants completed the questionnaire.

Participants were asked to imagine being employed in a big company, the *Smart & Tech Service*. This company has recently conducted a survey among employees in order to assess their opinion and impression about organizational life. We asked participants to read a fictitious extract of the final report about the internal investigation conducted by the company. In the *friendship condition*, participants read a paragraph in which employees described the ethical climate of the company in line with Victor and Cullen’s (1987) theorization of the friendship climate. So, participants read that the interviewed employees perceived an organizational climate in which importance was given to mutual well-being, respect for the rights of employees, taking care of the common good, and collective satisfaction. Moreover, the main objective of the employees was to achieve the objectives taking into account the interests of all the members of the group. By contrast, in the *self-interest condition*, participants read a paragraph in which employees described the ethical climate of the company in line with Victor and Cullen’s (1987) theorization of the self-interest climate. Thus, participants read that the interviewed employees perceived an organizational climate in which importance was given to productivity, self-assertion, the development of their skills, and career advancement. Furthermore, the main objective of the employees was to achieve their personal working goals and the protection of their interests. This manipulation was checked at the end of the questionnaire. Prior to the main study, we pretested the perception of the two scenarios in terms of valence to avoid any interference with the perception of the positiveness of the climate. Forty-one participants were recruited online *via* Qualtrics and were asked to randomly read one of the two scenarios and answer the following questions (19 females, 21 males, 1 unknown; mean age = 32.56; SD = 8.51): “The organizational climate of the described company is positive,” and “The employees of this company are satisfied” (from 1 = *completely disagree* to 6 = *completely agree*; *r*(39)* =* 0.80, *p* < 0.001). One participant was discarded because he or she did not complete the survey. In line with our expectation, an independent sample *t*-test confirmed that participants perceived the two companies as similarly positive [friendship condition: *M* = 4.69; SD = 1.00; self-interest condition: *M* = 4.18; SD = 1.24, *t*(38) = 1.43, *p* = 0.16, Cohen’s *d* = 0.45].

Participants were then asked to vividly imagine being an employee of the *Smart & Tech Service* and to complete a measure of *organizational identification*. We used the Italian adaptation by Manuti and Bosco (2012) of the original six-item scale by Mael and Ashforth (1992); e.g., “When someone criticizes my organization, it feels like a personal insult”; Cronbach’s alpha = 0.70. Subsequently, we assessed the extent to which the organization was perceived as moral by asking participants to indicate the extent to which three items described the organization (i.e., “honest,” “sincere,” and “trustworthy”; Cronbach’s alpha = 0.89; Leach et al., 2007).

Participants then completed the Italian version (Pierro et al., 1995) of the 20-item *organizational commitment* scale (e.g., “I would be very happy to spend the rest of my career with this organization”; “I enjoy discussing my organization with the people outside it”; Cronbach’s alpha = 0.81; Meyer and Allen, 1991). They then indicated their *turnover intention* (“If I had the opportunity, I would not think twice about changing jobs”), their *recommendation* of the organization (“I would recommend to a person close to me to apply for a possible job in the company”), and the *minimum wage* they would accept (“What is the minimum monthly salary that you would be willing to accept to work for the Smart and Tech Service?”; open measure) on single items. This last question was intended to tap participants’ extrinsic motivation to be part of the aforementioned organization.

Finally, to check the effectiveness of our manipulation, we asked participants to complete Cullen et al.’s (1993) measures of *Ethical organizational climate of self-interest* (four items; e.g., “In this company, people are mostly out for themselves”; Cronbach’s alpha = 0.86) and *Ethical organizational climate of friendship* (six items; e.g., “In this company, people look out for each other’s good”; Cronbach’s alpha = 0.94) in reference to the fictitious company they are part of. Two participants did not complete the manipulation checks, and their responses were eliminated from the data set (retained sample = 150). We ran the analyses with the whole sample, and the results were almost identical. In particular, the crucial mediation paths tested and reported below were still significant.

### Results

We conducted data analysis with IBM-SPSS 21.0 and JASP 0.9.0.1 statistical packages. Table 1 reports the descriptive statistics and zero-order correlations among the variables of the study. Differences in the degrees of freedom are due to instances of missing data. We performed an independent sample *t*-test for each dependent variable, with the *Ethical Climate* (friendship vs. self-interest) as a between-participants factor. Mediation analysis was performed through the regression approach and the bootstrap estimation through the adoption of PROCESS, the SPSS macro developed by Hayes and Preacher (2014).

**Table 1:** Study 1: Descriptive statistics and zero-order correlations on the whole sample among the variables of the study.

	*M* (SD)	1	2	3	4	5	6
1) Organizational identification	5.34 (0.90)	1.00	–	–	–	–	–
2) Organizational morality	5.41 (1.12)	0.40***	1.00	–	–	–	–
3) Commitment	4.44 (0.70)	0.49***	0.51***	1.00	–	–	–
4) Turnover intention	3.24 (1.51)	–0.03	–0.22**	–0.43***	1.00	–	–
5) Recommendation	5.51 (1.18)	0.38***	0.44***	0.60***	–0.25**	1.00	–
6) Minimum wage (in Euros)	1,709.52 (605.21)	–0.02	–0.12	–0.16^a^	0.02	–0.01	1.00


*^∗∗∗^p < 0.001, ^∗∗^*p* < 0.01, ^∗^*p* < 0.05, ^*a*^*p* = 0.05.*

#### Manipulation Checks

We first evaluated the effectiveness of the ethical climate manipulation. Participants assigned to the friendship condition (*M =* 2.84, SD = 1.32) reported lower levels of the ethical organizational climate of self-interest than those assigned to the self-interest condition [*M* = 4.46, SD = 1.45; *t*(148) = 7.10, *p* < 0.001, Cohen’s *d* = 1.16]. In an opposite direction, participants assigned to the friendship condition (*M =* 5.59, SD = 0.99) reported higher levels of the ethical organizational climate of friendship than those assigned to the self-interest condition [*M* = 3.78, SD = 1.35; *t*(148) = -9.30, *p* < 0.001, Cohen’s *d* = -1.52]. This confirms that our manipulation was effective.

#### Identification, Commitment, and Perception of Organizational Morality

In line with our hypotheses, participants assigned to the friendship condition identified more with the organization (*M =* 5.50, SD = 0.89) than those assigned to the self-interest condition [*M* = 5.19, SD = 0.88; *t*(148) = -2.11, *p* = 0.036, Cohen’s *d* = -0.35] and showed higher commitment toward the organization [*M*_Friendship_ = 4.69, SD = 0.67 vs. *M*_Self-Interest_ = 4.21, SD = 0.65; *t*(148) = -4.45, *p* < 0.001, Cohen’s *d* = -0.73]. Moreover, the organization was perceived as more moral in the friendship (*M =* 5.81, SD = 0.91) than in the self-interest condition [*M* = 5.06, SD = 1.17; *t*(146) = -4.30, *p* < 0.001, Cohen’s *d* = -0.71].

#### Turnover Intention, Recommendation, and Minimum Wage

Participants showed similar turnover intention in the friendship (*M =* 3.04, SD = 1.54) and in the self-interest condition [*M* = 3.42, SD = 1.47; *t*(148) = 1.52, *p* = 0.13, Cohen’s *d* = 0.25], although an inspection of the raw means seems to indicate a slightly higher intention to leave the organization in the latter condition, as we expected. Participants were also more willing to recommend to a person close to them to apply for a possible job in the company in the friendship condition (*M =* 5.93, SD = 0.90) than in the self-interest condition [*M =* 5.13, SD = 1.28, *t*(148) = -4.41, *p* < 0.001, Cohen’s *d* = -0.72]. Moreover, participants declared they would accept a lower salary (expressed in euros) to be part of the company characterized by an ethical climate of friendship (*M =* 1,575.00, SD = 466.23) than in the company characterized by an ethical climate of self-interest [*M =* 1,831.82 SD = 688.82, *t*(145) = 2.62, *p* = 0.01, Cohen’s *d* = 0.43].

Thus, an organization characterized by a friendship ethical climate seems to attract more individuals who identify with and commit to it and would be more willing to recommend the organization to a close friend and to accept a lower salary to be part of the company, as we hypothesized.

#### Mediation Analyses

We then conducted three mediation analyses to test whether the effect of ethical climate (coded as 0 = *self-interest*; 1 = *friendship*) on three main outcomes – that is, turnover intention, recommendation, and minimum wage – was mediated by organizational identification and commitment. We followed the procedure described by Hayes (2013) for estimating indirect effects. Organizational identification and commitment were modeled as sequential mediators (process model number 6), assuming that an ethical climate of friendship (vs. self-interest) would have elicited a stronger identification, and this, in turn, would have induced a stronger commitment to the organization. The resulting commitment would have then fostered the three outcomes. This order reflects also the order in which the constructs were assessed in the questionnaire.

##### Turnover intention

**FIGURE 1:**
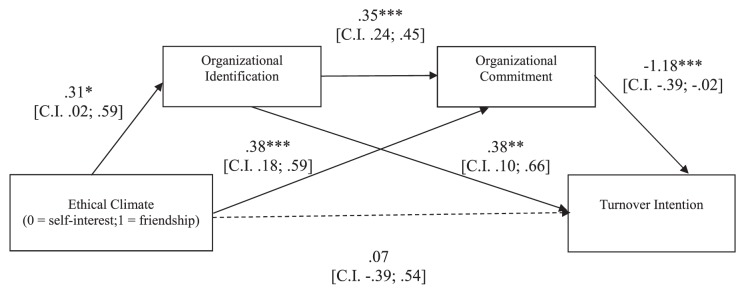
Study 1: Mediation model in which the effects of ethical climate of self-interest vs. friendship on participants’ turnover intention are mediated by organizational identification and commitment. ^∗^*p* < 0.05; ^∗∗^*p* < 0.01; ^∗∗∗^*p* < 0.001.

The overall equation was significant [*R*^2^ = 0.22, *F*(3,146) = 13.89, *p* < 0.001]. As shown in Figure 1, the ethical climate of friendship elicited a stronger organizational identification, and this, in turn, fostered a stronger commitment. Such commitment, then, made participants less willing to leave the organization. Supporting our hypothesis, a bootstrapping procedure with 5,000 resamples showed that the indirect effect of the experimental condition on participants’ turnover intention *through* the hypothesized causal chain was significant [*b* = -0.12, confidence interval (CI): lower limit (LL) = -0.30; upper limit (UL) = -0.02].

##### Recommendation

**FIGURE 2:**
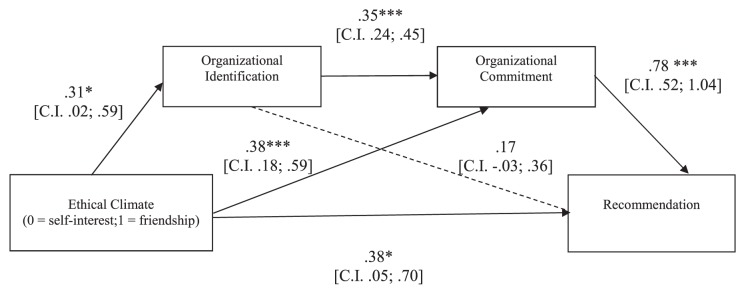
Study 1: Mediation model in which the effects of ethical climate of self-interest vs. friendship on participants’ recommendation are mediated by organizational identification and commitment. ^∗^*p* < 0.05; ^∗∗^*p* < 0.01; ^∗∗∗^*p* < 0.001.

The overall equation was significant [*R*^2^ = 0.37, *F*(3,146) = 29.04, *p* < 0.001]. As shown in Figure 2, in line with our hypothesis, the indirect effect of the experimental condition on participants’ recommendation *through* the hypothesized causal chain was significant (5,000 resampling; *b* = 0.08, CI: LL = 0.005; UL = 0.19), although even the direct effect of the ethical climate on recommendation remained significant. Thus, the effect of the ethical climate on recommendation resulted partially mediated by organizational identification and commitment.

##### Minimum wage

With regard to the minimum wage, although the overall equation was significant [*R*^2^ = 0.58, *F*(3,143) = 2.94, *p* < 0.05], the indirect effect was not reliable. Thus, the effect of ethical climate on the minimum wage participants would accept to be a part of the company was not explained by organizational identification and commitment.

### Discussion

On the overall, findings from Study 1 provided support to our main hypotheses. Individuals prefer more to be part of an organization characterized by an ethical climate of friendship, which subsumes a collective and interdependent way to manage ethical issues, than to an organization characterized by an ethical climate of self-interest, which stresses an individual and independent course of action. Moreover, in line with our rationale, this seems to reflect social identity concerns: In particular, organizational identification and the subsequent commitment seem to be crucial in determining the individual’s reactions to the different ethical climates. To further disentangle the primacy of an ethical climate of friendship as a way to ensure social identity motives and to encourage organizational commitment and positive behaviors, in Study 2, after assigning participants to fictitious organizations characterized by either a friendship or a self-interest ethical climate, we allowed them to evaluate a job offer stemming from an organization endorsing the opposite climate.

## Study 2

### Methods

#### Participants and Design

One hundred fifty-six participants completed an online questionnaire through the platform Qualtrics on a voluntary basis. Forty-three abandoned the survey without providing any responses. Thus, the final sample consisted of 113 participants (66 females, 36 males, 1 other, 10 unknown; mean age = 27.91; SD = 7.06). Like in Study 1, participants were randomly assigned to one of the two conditions, resulting in a between-participants design with one factor (*ethical climate*: friendship vs. self-interest).

#### Procedure

The procedure was largely identical to that of Study 1, with some relevant exceptions. As in the previous study, after providing their consent to take part in the research, participants were asked to imagine being employed in a big company, the *Smart & Tech Service*, characterized by either a friendship or a self-interest ethical climate. Participants then completed the same measures of Study 1: *organizational* *identification* (alpha = 0.85), *organizational morality* (alpha = 0.90), *organizational commitment* (alpha = 0.87), *turnover intention*, *recommendation of the organization*, and the *minimum wage* they would accept. As in Study 1, the manipulation of the two ethical climates was checked with Cullen et al.’s (1993) measures (*self-interest*, alpha = 0.84; *friendship*, alpha = 0.94).

Then, participants were asked to imagine themselves attending a meeting and being contacted by a second company (the “MassCom”) with a job offer. This new company was described as almost similar in terms of size and market position to the one the participants allegedly belong to. Nevertheless, this second company was described as characterized by the opposite perceived ethical climate: That is, in the friendship condition, it was described as embracing a self-interest ethical climate, and vice versa. Finally, we asked participants to indicate to what extent they would accept a job offer with the same salary and for the same role (*intention to switch organization*; from 1 = “absolutely not” to 7 = “absolutely yes”) and the *minimum wage* they would accept to transfer to the new company (open measure). They were then thanked and invited to contact the principal investigator for further information about the study.

### Results

We conducted data analysis with IBM-SPSS 21.0 and JASP 0.9.0.1 statistical packages. Table 2 reports the descriptive statistics and zero-order correlations among the variables of the study. Differences in the degrees of freedom are due to instances of missing data. Unless otherwise specified, we performed an independent sample *t*-test for each dependent variable, with the *ethical climate* (friendship vs. self-interest) as a between-participants factor.

**Table 2:** Study 2: Descriptive statistics and zero-order correlations on the whole sample among the variables of the study.

	*M* (SD)	1	2	3	4	5	6	7	8
1) Organizational identification	5.07 (1.04)	1.00	–	–	–	–	–	–	–
2) Organizational morality	5.11 (0.98)	0.49***	1.00	–	–	–	–	–	–
3) Commitment	4.30 (0.74)	0.71***	0.57***	1.00	–	–	–	–	–
4) Turnover intention	3.43 (1.33)	–0.40***	–0.26**	–0.53***	1.00	–	–	–	–
5) Recommendation	5.05 (1.21)	0.48***	0.45***	0.63***	–0.43***	1.00	–	–	–
6) Minimum wage– first company (in Euros)	1,765.53 (630.76)	–0.19*	–0.25**	–0.23*	0.18^a^	–0.13	1.00	–	–
7) Intention to switch	4.00 (1.51)	–0.39***	–0.22*	–0.48***	0.39***	–0.44***	0.08	1.00	–
8) Minimum wage – second company (in Euros)	2,161.51 (939.50)	0.05	–0.09	0.05	–0.09	0.12	0.63***	–0.25**	1.00


*^∗∗∗^p < 0.001, ^∗∗^*p* < 0.01, ^∗^*p* < 0.05, ^a^*p* = 0.05.*

#### Manipulation Checks

Participants assigned to the friendship condition (*M =* 3.14, SD = 1.22) reported lower levels of the ethical organizational climate of self-interest than those assigned to the self-interest condition [*M* = 4.84, SD = 1.27; *t*(107) = 7.13, *p* < 0.001, Cohen’s *d* = 1.37]. In an opposite direction, participants assigned to the friendship condition (*M =* 4.93, SD = 1.19) reported a higher level of the ethical organizational climate of friendship than those assigned to the self-interest condition [*M* = 3.45, SD = 1.37; *t*(107) = -6.01, *p* < 0.001, Cohen’s *d* = -1.16]. This confirms that our manipulation was again effective.

#### Identification, Commitment, and Perception of Organizational Morality

Participants assigned to friendship condition identified more strongly with the organization (*M =* 5.41, SD = 0.87) than those assigned to the self-interest condition [*M* = 4.66, SD = 1.09; *t*(111) = -4.02, *p* < 0.001, Cohen’s *d* = -0.76] and showed a higher commitment toward the organization [*M*_Friendship_ = 4.53, SD = 0.57 vs. *M*_Self-Interest_ = 4.01, SD = 0.83; *t*(111) = -3.99, *p* < 0.001, Cohen’s *d* = -0.76]. Moreover, the organization was perceived as more moral in the friendship (*M =* 5.31, SD = 0.87) than in the self-interest condition [*M* = 4.85, SD = 1.06; *t*(111) = -2.49, *p* = 0.014, Cohen’s *d* = -0.47]. Confirming Study 1, when the ethical climate promotes interdependence and collectivism rather than independence and individualism, individuals identify more strongly with an organization, commit to it, and perceive it as more moral.

#### Turnover Intention, Recommendation, and Minimum Wage

Participants showed a higher intention to leave the organization in the self-interest condition (*M =* 3.74, SD = 1.37) than in the friendship condition [*M* = 3.19, SD = 1.27; *t*(111) = 2.21, *p* = 0.03, Cohen’s *d* = 0.42], in line with our prediction. Participants were more willing to recommend to a person close to them to apply for a possible job in the company in the friendship condition (*M =* 5.48, SD = 0.97) than in the self-interest condition [*M =* 4.52, SD = 1.28), *t*(111) = -4.53, *p* < 0.001, Cohen’s *d* = -0.86]. Moreover, although the effect was not reliable, participants declared they would accept a slightly lower salary (expressed in euros) to be part of the company characterized by an ethical climate of friendship (*M =* 1,675.81, SD = 338.48) than in the company characterized by an ethical climate of self-interest [*M =* 1,878.57 SD = 860.83, *t*(111) = 1.71, *p* = 0.09, Cohen’s *d* = 0.32].

#### Intention to Switch Organization

We then tested participants’ intention to leave their organization, joining a different one that embraces the opposite ethical climate. In line with Hp4, participants assigned to the self-interest condition exhibited higher intention to leave their organization and accepting a job offer from an organization characterized by an ethical climate of friendship (*M =* 4.94, SD = 1.20) than participants in the friendship condition [*M* = 3.25, SD = 1.31; *t*(111) = 7.05, *p* < 0.001, Cohen’s *d* = 1.34].

#### Minimum Acceptable Wage in the New Organization

In order to test our Hp5, we performed a 2 (*ethical climate*: friendship vs. self-Interest) by 2 (*minimum acceptable wage*: old organization vs. new organization) mixed-model analysis of variance (ANOVA), with the last factor varying within participants. The main effect of ethical climate was not reliable [*F*(1,111) = 0.01, *p* = 0.91]. By contrast, the main effect of the minimum acceptable wage was significant [(*F*(1,111) = 30.78, *p* < 0.001, partial η^2^ = 0.22], indicating that participants asked for a higher minimum wage to work for the new organization (*M =* 2,161.51, SD = 939.50) than for the old one (*M =* 1,765.53, SD = 630.76). This effect was qualified by a reliable two-way interaction [*F*(1,111) = 7.74, *p* = 0.006, partial η^2^ = 0.07]. To disentangle this interaction, we performed a simple main effect analysis. Participants assigned to the friendship condition asked for a significantly higher wage to join an organization characterized by an ethical climate of self-interest (*M =* 2,237.98, *SE* = 118.39) compared to the minimum acceptable wage they indicated to be part of their own organization (*M =* 1,675.81, *SE =* 78.79), *F*(1,111) = 39.21, *p* < 0.001, partial η^2^ = 0.26. Participants assigned to the self-interest condition asked for a marginally higher wage to join an organization characterized by an ethical climate of friendship (*M =* 2,065.13, SE = 132.90) compared to the minimum acceptable wage they indicated to be part of their own organization (*M =* 1,878.57, SE* =* 88.44), *F*(1,111) = 3.43, *p* = 0.07, partial η^2^ = 0.03. In line with Hp5, participants exposed to an ethical climate of friendship, compared to those exposed to an ethical climate of self-interest, asked for more money in order to transfer to the new organization characterized by the opposite climate.

#### Mediation Analyses

As in Study 1, we then conducted sequential mediation analyses to test whether the effect of ethical climate (coded as 0 = *self-interest*; 1 = *friendship*) on the main outcomes – that is, turnover intention, recommendation, minimum acceptable wage relative to the old and the new company, and intention to switch organization – was mediated by organizational identification and commitment.

##### Turnover intention

**FIGURE 3:**
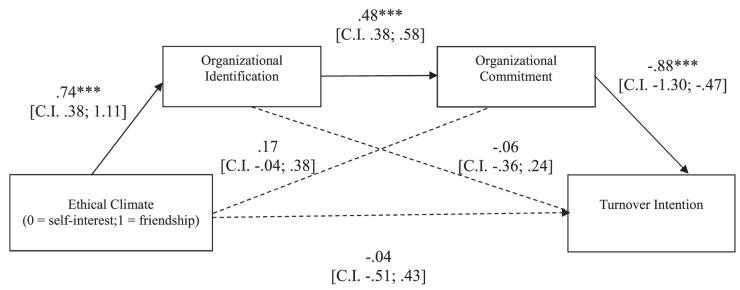
Study 2: Mediation model in which the effects of ethical climate of self-interest vs. friendship on participants’ turnover intention are mediated by organizational identification and commitment. ^∗^*p* < 0.05; ^∗∗^*p* < 0.01; ^∗∗∗^*p* < 0.001.

The overall equation was significant [*R*^2^ = 0.28, *F*(3,109) = 14.21, *p* < 0.001]. As shown in Figure 3, the ethical climate of friendship elicited a stronger organizational identification, and this, in turn, fostered a stronger commitment. Such commitment, then, made participants less willing to leave the organization. Supporting our hypothesis, a bootstrapping procedure with 5,000 resamples showed that the indirect effect of the experimental condition on participants’ turnover intention *through* the hypothesized causal chain was significant (*b* = -0.31, CI: LL = -0.62; UL = -0.09).

##### Recommendation

**FIGURE 4:**
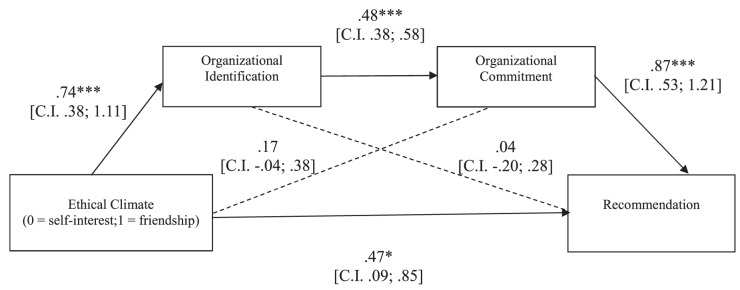
Study 2: Mediation model in which the effects of ethical climate of self-interest vs. friendship on participants’ recommendation are mediated by organizational identification and commitment. ^∗^*p* < 0.05; ^∗∗^*p* < 0.01; ^∗∗∗^*p* < 0.001.

The overall equation was significant [*R*^2^ = 0.43, *F*(3,109) = 26.93, *p* < 0.001]. As shown in Figure 4, in line with our hypothesis, the indirect effect of the experimental condition on participants’ recommendation *through* the hypothesized causal chain was significant (5,000 resampling; *b* = 0.31, CI: LL = 0.12; UL = 0.57), although even the direct effect of the ethical climate on recommendation remained significant. Thus, the effect of the ethical climate on recommendation resulted partially mediated by organizational identification and commitment.

##### Intention to switch organization

**FIGURE 5:**
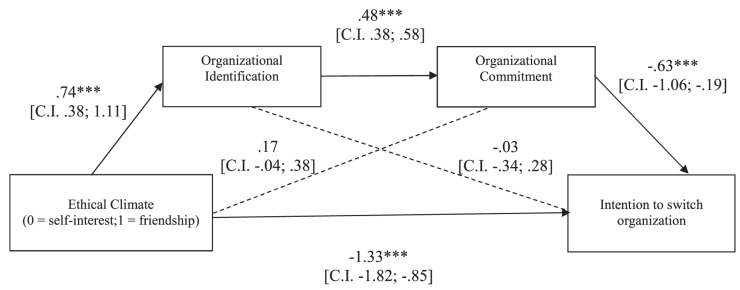
Study 2: Mediation model in which the effects of ethical climate of self-interest vs. friendship on participants’ intention to switch organization are mediated by organizational identification and commitment. ^∗^*p* < 0.05; ^∗∗^*p* < 0.01; ^∗∗∗^*p* < 0.001.

The overall equation was significant [*R*^2^ = 0.40, *F*(3,109) = 24.27, *p* < 0.001]. As shown in Figure 5, in line with our hypothesis, the indirect effect of the experimental condition on participants’ intention to move to an organization characterized by the opposite ethical climate *through* the hypothesized causal chain was significant (5,000 resampling; *b* = -0.22, CI: LL = -0.52; UL = -0.04), although even the direct effect of the ethical climate remained significant. Thus, the effect of the ethical climate on intention to switch organization resulted partially mediated by organizational identification and commitment.

##### Minimum acceptable wage

Concerning the minimum wage relative to the old and the new company, the mediation analyses were not reliable. In both cases, the overall equation did not reach significance [*R*^2^ = 0.06, *F*(3,109) = 2.35, *p =* 0.08; and *R*^2^ = 0.01, *F*(3,109) = 0.32, *p =* 0.81, respectively], and the indirect effect was not reliable. Again, the effect of ethical climate on the minimum wage participants would accept to be part of the company and/or to move to a different company characterized by the opposite climate was not explained by organizational identification and commitment.

## General Discussion

Ethical climate is a fundamental aspect of organizational life, which directly influences both individual and organizational outcomes and behaviors. As a group regulation tool, it represents a core antecedent of employees’ emotions, perceptions, and behaviors. Indeed, researchers so far confirmed that ethical climate may be predictive of both positive and negative employees’ attitudes and behaviors. This is why it is important to understand its effect in order to promote positive work behaviors and, on the contrary, to prevent or discourage deviant work behaviors.

In the present paper, we advanced that different ethical climates may differently predict employees’ attitudes and behaviors, depending on whether they embrace an individualistic and independent or a collectivistic and interdependent way to manage ethical issues within organizations. This is why we experimentally compared the effect of two kinds of climate derived from the literature: An ethical climate of self-interest and an ethical climate of friendship. This choice was theoretically grounded in the social identity approach to the study of organizational processes. Although previous research provided preliminary evidence for a differential association between self-interest vs. friendship, ethical climate, and employees’ attitudes and behaviors (Pagliaro et al., 2018), empirical support for a causal effect was still lacking. For this reason, we adopted an experimental approach by examining individual’s reactions to fictitious scenarios describing organizations characterized by either a self-interest or a friendship ethical climate. This allowed us to disentangle how an ethical climate of friendship predicts better attitudes and behavioral intentions among employees than an ethical climate of self-interest does. Crucially, building on the social identity main statements, we were able to demonstrate that organizational identification plays a crucial role in leading the effects of different ethical climates. In fact, the ethical climate of friendship fosters stronger identification with (and commitment to) the organization, which, in turn, promotes pro-organizational behavior (e.g., recommendation) and discourages negative tendencies (e.g., turnover intention). On the contrary, organizational identification diminished in the case of a self-interest climate, and this promoted intention to leave the organization. An interesting result emerged in both the studies presented here with regard to the minimum wage that the participants would have accepted to be part of the organization. On the one hand, it emerged that people asked for more money when they belong to an organization characterized by an ethical climate of self-interest (vs. friendship). On the other hand, Study 2 provided evidence that people belonging to a friendship organization asked for more money to move to a self-interest one. This evidence seems to indicate that whereas individuals may join, identify with, and commit to friendship organizations for an intrinsic and symbolic motivation, they may join self-interest ones for a more extrinsic motivation, such as the salary. So, overall, our research contributes to the literature pointing out the fundamental role of ethical climate as an organizational regulation tool, demonstrating that employees react differently to the different ways organizations act in order to manage ethical issues.

## Practical Implications

Recent developments in the global economic situation and in organizations emphasize both the centrality of work ethics and the negative impact of unethical behavior on workers, consumers, and other stakeholders (Grisaffe and Jaramillo, 2007; Tanner et al., 2015; Barattucci et al., 2017). The understanding and management of ethical climate, from an operational point of view, become on the one hand an opportunity to invest in corporate identification processes and, on the other hand, a potential to prevent and manage critical phenomena (CWB, moral disengagement, mistrust, psychological contract breach, etc.) with related costs to be faced (Peterson, 2002; Neubert et al., 2009; Newman et al., 2017). Since ethical climate has a considerable influence on behaviors and attitudes, as our studies further illustrated, organizations should monitor this so as to be in constant alignment with corporate strategies (Elçi and Alpkan, 2009; Arnaud and Schminke, 2012). As well as monitoring actions, HRM should provide ethical climate reinforcement practices, through management of organizational identification processes, through empowerment and communication processes (e.g., diffusion of code of ethics, activities, debates, discussions, workgroups, quality certification, etc.), through a careful administration of performance assessment and diversity management processes, and through specific training plans for leaders (e.g., Shin, 2012; Mayer, 2014; Pietroni and Hughes, 2016; Ning and Zhaoyi, 2017; Sartori et al., 2018). The ability to create an ethical climate consistent with corporate policies and objectives is a primary objective, a sort of need for the successful company that intends to use the high levels of trust of its workers as an economic and relational driver, with a deep effect also on relations with customers and partners. In order to activate this virtuous circle between climate and performance, it is necessary to intervene on the credibility of the company, through an initial process of communication and declaration of intent (to identify the objectives and the value creation process); subsequently, the company must concentrate on demonstrating ethical concerns in dealing with its collaborators and partners and increasing its credibility. It is not sufficient just to declare that an ethical climate analysis is necessary and needs to be monitored; rather, the company must also implement the best communication strategies for delivering ethical climate analysis results and prepare future lines of investigation.

The present results offer then further insights at a practical level. For instance, understanding how different ethical climates foster organizational identification may help to focus interventions targeting specific dimensions of organizational climate in order to increase positive organizational identification and, as a distal consequence, to promote pro-organization behavior. On the other hand, by showing that an ethical climate based on self-interest, individualism, and competitiveness reduced identification, we focused practitioners’ attention to the recognition of individualistic tendencies within organizations.

## Limitations and Future Directions

One of the main strength of the present paper is represented by the experimental approach we adopted to disentangle the causal link between ethical climates and their consequences. This required us to create *ad hoc*, fictitious scenarios describing organizations characterized by different ethical climates. Thus, a limitation of the present set of studies is relative to the fact that participants were asked to imagine a fictitious situation, instead of responding to a real context. Nevertheless, respondents have interpreted the experimental situation we have designed as we intended, as witnessed by both the pilot study and the manipulation checks. Indeed, experimental procedures are increasingly adopted in the organizational field (Haslam, 2004). Moreover, the causal effects we highlighted mirrored the correlational ones that emerge in a real context (Pagliaro et al., 2018).

Future research may be directed to investigate whether the role individuals occupy within the organization or specific features of the organization itself (e.g., market position, size, and so on) moderate the pattern of results highlighted here. Moreover, many studies have investigated the relationship between styles and types of leadership and ethical climate (e.g., Brown et al., 2005; Ning and Zhaoyi, 2017). Based on what we found here, ethical leadership seems to be fundamental to implementing an ethical climate, since when leaders demonstrate ethical behavior, employees will most frequently follow ethical expectations (Zehir et al., 2014). In our future agenda, therefore, it will be necessary to consider leadership variables (e.g., distributed or transformational leadership) and their relationship to climate.

To conclude, with the present research, we extended our knowledge on the dynamics of ethical climate and further grounded our understanding of such dynamics in a widespread theoretical approach, such as the one represented by social identity.

## Data Availability

The datasets generated for this study are available on request to the corresponding author.

## Ethics Statement

We declare that the experiments have been conducted in line with ethical standards of the 1964 Declaration of Helsinki. Before taking part in the experiments, participants were informed about any relevant aspect of the study (e.g., methods, institutional affiliations of the researcher) and they were informed of the right to refuse to participate in the study or to withdraw consent to participate at any time during the study without reprisal. They then confirmed that they well understood the instructions, accepted to participate, and filled in the questionnaire. Once participants completed their questionnaires they were thanked, fully debriefed, and asked to sign a further consent form to use their data for scientific purposes.

## Author Contributions

All the authors developed the present research and equally contributed to this manuscript. MT collected and analyzed the data.

## Conflict of Interest Statement

The authors declare that the research was conducted in the absence of any commercial or financial relationships that could be construed as a potential conflict of interest.
